# A new weighting factor in combining belief function

**DOI:** 10.1371/journal.pone.0177695

**Published:** 2017-05-25

**Authors:** Deyun Zhou, Qian Pan, Gyan Chhipi-Shrestha, Xiaoyang Li, Kun Zhang, Kasun Hewage, Rehan Sadiq

**Affiliations:** 1 School of Electronics and Information, Northwestern Polytechnical University, Xi’an, China; 2 School of Engineering, University of British Columbia Okanagan, Kelowna, BC, Canada; Southwest University, CHINA

## Abstract

Dempster-Shafer evidence theory has been widely used in various applications. However, to solve the problem of counter-intuitive outcomes by using classical Dempster-Shafer combination rule is still an open issue while fusing the conflicting evidences. Many approaches based on discounted evidence and weighted average evidence have been investigated and have made significant improvements. Nevertheless, all of these approaches have inherent flaws. In this paper, a new weighting factor is proposed to address this problem. First, a modified dissimilarity measurement is proposed which is characterized by both distance and conflict between evidences. Second, a measurement of information volume of each evidence based on Deng entropy is introduced. Then two kinds of weight derived from aforementioned measurement are combined to obtain a new weighting factor and a weighted average method based on the new weighting factor is proposed. Numerical examples are used to illustrate the validity and effectiveness of the proposed method. In the end, the new method is applied to a real-life application of river water quality monitoring, which effectively identify the major land use activities contributing to river pollution.

## Introduction

Dempster-Shafer (D-S) evidence theory provides a reasonable and efficient way to deal with the information which is uncertain and discordant. It has been extensively used in various applications related to decision-making such as information fusion [1–3], uncertain reasoning [4], fault diagnosis [5], risk analysis [6–9], cognitive map [10], target recognition and association [11–13]. Unlike the probability theory and Bayesian theory, the D-S evidence theory requires few prior conditions and knowledge when information is processed. For example, the Evidential Reasoning (ER) algorithm is a generalized Bayesian inference process and the ER rule reveals that the combined degree of joint support for a proposition from two pieces of independent evidence constitutes two parts in general [14–16]. When there is no priori information, the ER rule will reduce to the D-S combination rule. Moreover, the combination rule of the D-S evidence theory satisfies some of mathematical properties, such as commutativity and associativity. However, counter-intuitive results may occurred by the normalization step of the classical D-S combination rule when collected sources of evidence highly conflict with each other, as pointed out by Zadeh [17]. The effectiveness of the D-S evidence theory will be considerably reduced by this deficiency.

Evidently, it is crucial to handle the evidences with high conflict. In the last few years, many researchers have carried out comprehensive research and have applied a series of modifications to the conventional evidence combination rule [18–24]. In general, the existing methods can be divided into two kinds of solutions: revisal of the combination rule and revisal of the evidences, for the problem of high level conflict evidence fusion. Proponents of the first argument present that illogical results are caused by inappropriate distribution of the conflict information. Therefore, the modified methods based on the revisal of the combination rule mainly focus on altering the assignment of conflict information [2, 18, 21, 25–27]. Among them, solutions of the transferable belief model (TBM) and Dezert-Smarandache theory (DSmT) are more popular. The TBM develops a method to transfer the basic belief assignments (BBAs) to probabilities, but the method only can be used in closed world [25, 28]. DSmT extends the assignment universe of BBAs from a power set to a super power set which is more thorough and complete, and corresponding combination models and rules are developed as well [27]. Nonetheless, the modified combination rules have limitations in some situation. For example, most of the modified combination rules are not commutative and associative and are time consuming when dealing with a large amount of evidences.

The other modification, revisal of the evidences, preprocesses conflict evidences before combination process. The favorable mathematical properties of the D-S combination rule are reserved in the improvement as they do not change the D-S combination rule. Many related work have been proposed to support this modification method [22–24, 29–32]. Murphy [29] generates a new evidence by averaging *N* evidences with equal weights and then combine it with *N*-1 times. Based on this idea, Deng [22] proposes a weighted averaging method to obtain the new evidence. Besides the weighted averaging method, the discounting method also plays an important role in preprocessing conflict evidences. The weighting factor of both the weighted average method and discounting method can be identified by evidence distance, which is usually used to describe the conflict or dissimilarity [33–35]. Liu [35] argues that the conflict coefficient *k* in the evidence theory is inadequate to reflect the degree of conflict and dissimilarity between evidences, and he utilizes a two-dimension cell <*evidence conflict*, *evidence distance*> to measure the dissimilarity between evidences. The cell is indeed more comprehensive and adequate than the single coefficient *k* when describing the dissimilarity, but it also has intrinsic shortcomings in practical situation. For instance, the conflict tolerance threshold *ε* is largely subjective and depends on the perception of a decision maker. In addition, the cell is not syncretized in the combination rule. A dissimilarity measure is proposed on the basis of Hamacher T-conorm fusion rules given by Liu, who considers not only the evidence conflict and distance but also combines it in the combination rule as a discount [36]. Nevertheless, there are twofold limitations associated with the mathematical modeling. First, since the conflict factors only use the maximal subjective probability of the BBAs, it cannot solve the situation related to the propositions with equal belief values, which are investigated in Section 3. Second, combining the evidence one by one has a low convergence rate.

Besides evidence conflict and distance, the evidence volume is another criterion to measure the importance of an evidence [37]. If an evidence has more information, it should have a greater impact on the final aggregated result. Deng entropy [38], as a generalization of Shannon entropy, can measure the evidence information volume under the framework of D-S evidence theory. In this paper, Deng entropy and modified dissimilarity measure are used to form a new weighting factor. Then the new combination rule of evidence is carried out based on the new weighting factor, which has improved the versatility and has a fast convergence rate.

This paper is organized as follows. Section 2 describes some basic concepts related to the D-S evidence theory and dissimilarity measure. Section 3 presents problems of existing conflict coefficients, especially the limitations of Liu’s method. Section 4 investigates the new weighting factor of modified dissimilarity and Deng entropy, and some examples and analysis are presented to show the superiority and effectiveness of proposed method. In Section 5, the proposed method is used in a real-life application of the identification of water pollution sources. Finally, conclusions are drawn in Section 6.

## Preliminaries

### 2.1 Basics of D-S evidence theory

*Definition 1*. Suppose *Θ* be a nonempty finite set of mutually exclusive alternatives and defined as frame of discernment. Set of all the possible subsets of *Θ*, denoted by 2^*Θ*^, is called power set. The mapping *m*: 2^*Θ*^ → [0,1] is defined as the basic belief assignment (BBA) (also known as basic probability assignment, BPA) [39, 40]. The BBA satisfies

$$\sum_{A\subseteq \Theta }m(A)=1$$ (1)

m(∅)=0 (2)

where *m*(*A*) reflects the strength of each of evidence support for the proposition *A* in the frame of discernment, and ∅ denotes the empty set of *Θ*. *A* is called the focal element, if *m*(*A*) > 0.

*Definition 2*. The belief function *Bel*(*A*) and plausibility function *Pl*(*A*) from a BBA are defined as

$$Bel(A)=\sum_{B\subseteq A}m(B)$$ (3)

$$Pl(A)=\sum_{B\cap A\neq \emptyset }m(B)$$ (4)

where *Bel*(*A*) represents the amount of belief that definitely support *A*, and the *Pl*(*A*) could be viewed as the amount of belief that potentially placed in *A*.

*Definition 3*. Let *m*_1_ and *m*_2_ be two BBAs defined on the same frame *Θ*. D-S evidence theory combination rule is expressed as

$$m(A)=\left\{ \begin{array}{l} \frac{\sum_{B\cap C=A}m_{1}(B)m_{2}(C)}{1-k}\;A\neq \emptyset \\ \;0\;A=\emptyset \end{array}\right.$$ (5)

with

$$k=\sum_{B\cap C=\emptyset }m_{1}(B)m_{2}(C)$$ (6)

where *k* is named as conflict coefficient to measure the degree of conflict between two BBAs. The combination rule is out of work when *k* = 1.

Zadeh [17] presents a famous example that the D-S combination rule will produce an unexpected result. Suppose a frame is *Θ* = {*A*,*B*,*C*} and two BBAs are given as

$$m_{1}:m_{1}{(A)=0.99,m}_{1}(B)=0.01$$

$$m_{2}:m_{2}(B)=0.01,m_{2}(C)=0.99$$

by the D-S combination rule, the aggregated result is *k* = 0.9999, *m*(*A*) = *m*(*C*) = 0 and *m*(*B*) = 1, which is obviously counter-intuitive and unreasonable.

### 2.2 Jousselme distance

Jousselme distance [32], considering both the mass and cardinality of focal elements of each BBA, is commonly used as the measure of dissimilarity.

*Definition 4*. Let *m*_1_ and *m*_2_ be two BBAs on the same frame *Θ*, containing *N* mutually exclusive and exhaustive propositions. The Jousselme distance between *m*_1_ and *m*_2_ are defined as

$${d_{J}}_{m_{2}}^{m_{1}}=\sqrt{0.5*({\left\|m_{1}\right\|}^{2}+{\left\|m_{2}\right\|}^{2}-2\langle {m_{1},m}_{2}\rangle )}$$ (7)

where ‖*m*_1_‖^2^ = 〈*m*_1_,*m*_1_〉, ‖*m*_2_‖^2^ = 〈*m*_2_,*m*_2_〉 and 〈*m*_1_,*m*_2_〉 is given by

$$\langle {m_{1},m}_{2}\rangle =\sum_{i=1}^{2^{N}}\sum_{j=1}^{2^{N}}m_{1}(A_{i})m_{2}(B_{j})\frac{\left|A_{i}\cap B_{j}\right|}{\left|A_{i}\cup B_{j}\right|}$$ (8)

with *A*_*i*_ and *B*_*j*_ are the elements of the power set 2^*Θ*^. |*A*_*i*_ ∩ *B*_*j*_| and |*A*_*i*_ ∪ *B*_*j*_| denote the cardinality intersection set and union set of *A*_*i*_ and *B*_*j*_.

### 2.3 Probabilistic-based distance

Since the probalilistic transformation has an ability to convert a BBA from the focal elements into a probability measure of distinct atomic, it provides a probabilistic-based distance to measure the dissimilarity of two evidences [41].

*Definition 5*. Let *m* be a BBA on a frame *Θ*, and the probabilistic expression of a singleton element *B* in *Θ* could be obtained by pignistic probability function

$${BetP}_{m}(B)=\sum_{A\in 2^{\Theta },B\subseteq A}\frac{m(A)}{\left|A\right|}$$ (9)

where |*A*| is the cardinality of proposition *A*. If |*A*| = 1, then *B* = *A* and *BetP*(*B*) = *BetP*(*A*) = *m*(*A*).

*Definition 6*. Let *m*_1_ and *m*_2_ be two BBAs on the same frame *Θ* and let ${BetP}_{m_{1}}$ and ${BetP}_{m_{2}}$ be the results of pignistic probability transformation of *m*_1_ and *m*_2_, the probabilistic-based distance ${difBetP}_{m_{1}}^{m_{2}}$ is defined as

$${difBetP}_{m_{1}}^{m_{2}}=\max_{A\in \Theta }(|{BetP}_{m_{1}}(A)-{BetP}_{m_{2}}(A)|)$$ (10)

and the Murkowski distance [36] proposed by Liu is defined as

$${distP}_{m_{1}}^{m_{2}}=\sum_{A_{i}\in \Theta }0.5*(\left|{BetP}_{m_{1}}(A_{i})-{BetP}_{m_{2}}(A_{i})\right|)$$ (11)

### 2.4 Combinatorial dissimilarity measure

Some compound dissimilarity measures are presented based on the conflict coefficient, evidence distance and probabilistic-based distance.

*Definition 7*. Let *m*_1_ and *m*_2_ be two BBAs on the same frame *Θ*, and a combinatorial dissimilarity measure based on the conflict coefficient $k_{m1}^{m2}$ and probabilistic-based distance ${difBetP}_{m_{1}}^{m_{2}}$ is defined as

$${cf}_{m_{2}}^{m_{1}}=\langle {k_{m1}^{m2},difBetP}_{m_{1}}^{m_{2}}\rangle$$ (12)

*m*_1_ and *m*_2_ are in conflict, iff both $k_{m1}^{m2}>\varepsilon$ and ${difBetP}_{m_{1}}^{m_{2}}>\varepsilon$. *ε* ∈ [0,1] denotes the threshold of conflict tolerance, and identified according to different applications [35].

*Definition 8*. Let *m*_1_ and *m*_2_ be two BBAs on the same frame *Θ*, and a combinatorial dissimilarity measure [42] based on the conflict coefficient $k_{m1}^{m2}$ and Jousselme distance ${d_{J}}_{m_{1}}^{m_{2}}$ is defined as

$$k_{d}=\frac{1}{2}\times \left(k_{m1}^{m2}+{d_{J}}_{m_{1}}^{m_{2}}\right)$$ (13)

*Definition 9*. Let *m*_1_ and *m*_2_ be two BBAs on the same frame *Θ* and let ${BetP}_{m_{1}}$ and ${BetP}_{m_{2}}$ be the results of pignistic probability transformation of *m*_1_ and *m*_2_. Then a combinatorial dissimilarity measure based on the Hamacher T-conorm fusion rules [43] is defined as

$${DismP}_{m1}^{m2}≜T\left({distP}_{m_{1}}^{m_{2}},{ConfP}_{m_{1}}^{m_{2}}\right)=\frac{{distP}_{m_{1}}^{m_{2}}+{ConfP}_{m_{1}}^{m_{2}}}{1+{distP}_{m_{1}}^{m_{2}}∙{ConfP}_{m_{1}}^{m_{2}}}$$ (14)

where ${ConfP}_{m_{1}}^{m_{2}}$ denotes the conflict coefficient based on the pignistic probability,

$${ConfP}_{m_{1}}^{m_{2}}=\left\{ \begin{array}{l} \;0,\;if\;BetP(X_{m_{1}}^{max})\cap {BetP(X}_{m_{2}}^{max})\neq \emptyset \\ BetP(X_{m_{1}}^{max})∙{BetP(X}_{m_{2}}^{max}),\;otherwise \end{array}\right.$$ (15)

where $BetP(X_{m_{1}}^{max})=arg\;\max_{x\in \Theta }{BetP}_{m_{i}}(x),i=1,2$

### 2.5 Deng entropy

Deng entropy, as a generalization of Shannon entropy, provide a solution to measure the information volume of a BBA. It is observed that the Deng entropy and Shannon entropy correspond to an uncertain degree of measurement [38].

*Definition 10*. Let *m* be a BBA on the frame *Θ* and the Deng entropy of *m* is defined as

$$E_{d}=-\sum_{i}m(A_{i})log\frac{m(A_{i})}{2^{\left|A_{i}\right|}-1}$$ (16)

where *A*_*i*_ is a proposition in BBA *m*, and |*A*_*i*_| is the cardinality of *A*_*i*_. The Deng entropy will become identical to Shannon entropy if |*A*_*i*_| = 1, that is

$$E_{d}=-\sum_{i}m(A_{i})log\frac{m(A_{i})}{2^{\left|A_{i}\right|}-1}=-\sum_{i}m(A_{i})logm(A_{i})$$ (17)

## Limitations of exiting dissimilarity measurements between BBAs

*Example 1*. Let *m*_1_, *m*_2_ and *m*_3_ be three BBAs on the same frame *Θ* with four propositions *Θ* = {*A*_1_,*A*_2_,*A*_3_,*A*_4_}. The three BBAs are given as

$$m_{1}:\;m_{1}(A_{1})=m_{1}(A_{2})=m_{1}(A_{3})=m_{1}(A_{4})=0.25$$

$$m_{2}:\;m_{2}(A_{1})=m_{2}(A_{2})=m_{2}(A_{3})=m_{2}(A_{4})=0.25$$

$${m_{3}:\;m}_{3}(A_{1})=m_{3}(A_{2})=m_{3}(A_{3})=1/3$$

we can get the conflict coefficients $k_{m_{1}}^{m_{2}}=0.75$ and $k_{m_{1}}^{m_{3}}=0.67$ by using Eq (6) between the BBAs. The result shows that the degree of conflict between *m*_1_ and *m*_2_ is bigger than the degree of conflict between *m*_1_ and *m*_3_, and they both are in relative high conflict. In fact, there is no conflict intuitively between *m*_1_ and *m*_2_ because they are the same. By using the Eq (10) and Eq (12), we can get the ${difBetP}_{m_{1}}^{m_{2}}=0$ and *cf*(*m*_1_,*m*_2_) = 〈0.75,0〉, which illustrates that *m*_1_ and *m*_2_ are consistent and measurement of coefficient *k* cannot measure the degree of conflict between the evidences in this situation. Although the combined measurement implies that the D-S combination rule should be used, it cannot conclude that how much the error will be conduct by using the combination rule. Therefore, the combination rule has a limitation in terms of providing an explicit expression and cannot be used directly in the combination rule.

*Example 2*. Let *m*_1_ and *m*_2_ be two BBAs on the same frame *Θ* = {*A*_1_,*A*_2_,…,*A*_2*n*_}, such that

$$m_{1}:\;m_{1}(A_{1})=m_{1}(A_{2})=\cdots =m_{1}(A_{n})=\frac{1}{n}$$

$$m_{2}:\;m_{2}(A_{n+1})=m_{2}(A_{n+2})=\cdots =m_{2}(A_{2n})=\frac{1}{n}$$

**Fig 1:**
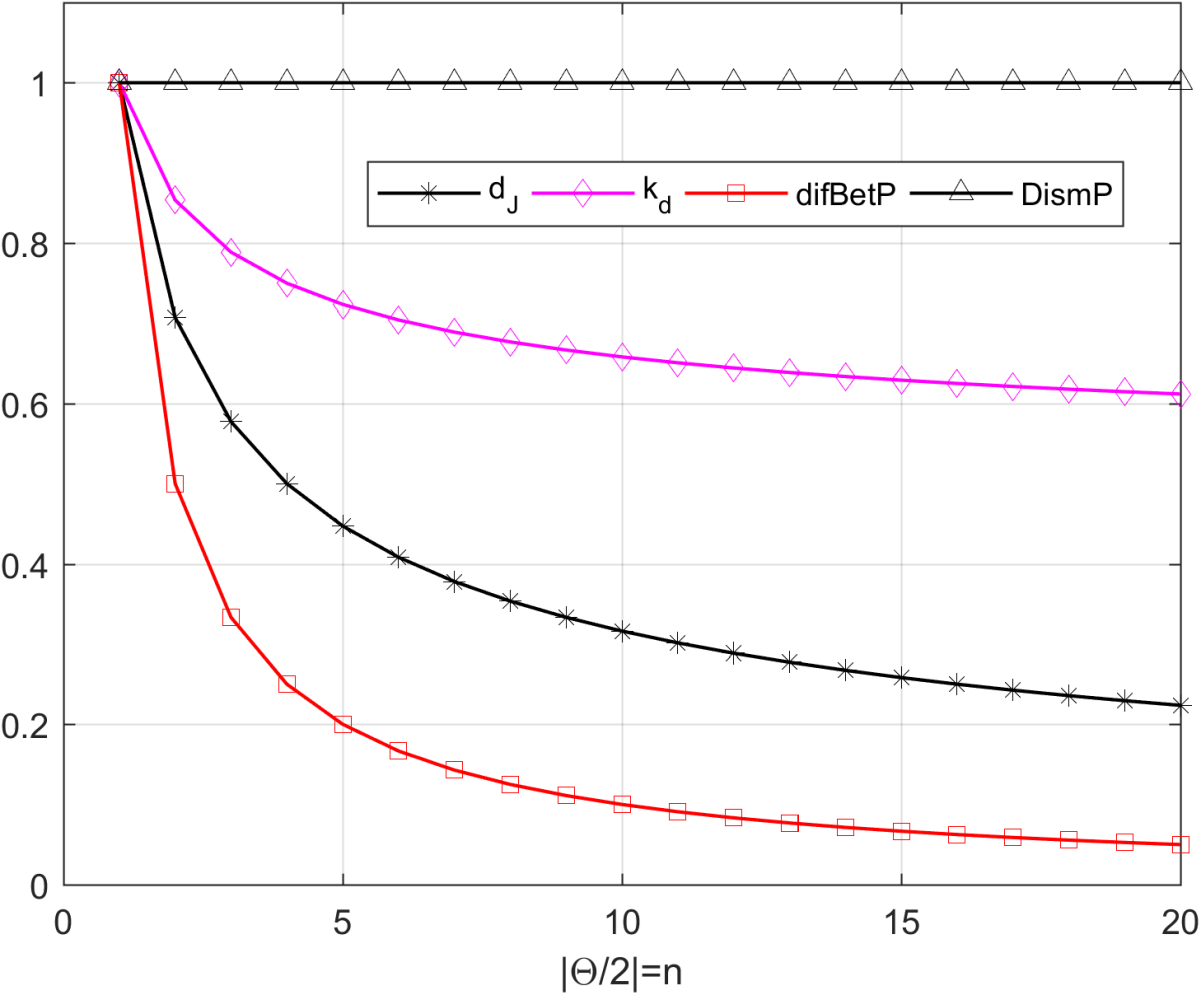
Different dissimilarity measurements.

It is obvious that the *m*_1_ and *m*_2_ are totally contrary to each other as they support the different propositions. The different dissimilarities between *m*_1_ and *m*_2_ are displayed in Fig 1.

From Fig 1, it is evident that the values of the *d*_*J*_, *k*_*d*_, and *difBetP* are 1, when *n* = 1, which are intuitive. But when *n* > 1, the values of *d*_*J*_ and *difBetP* tend to 0, and *k*_*d*_ tends to 0.6, indicating that *m*_1_ and *m*_2_ are getting closer and less conflict with the increase of *n*, which are counter-intuitive and abnormal. Only the *DismP* keeps 1 with the increase with *n*, meaning that the *m*_1_ and *m*_2_ are totally in disagreement with each other. Therefore, *d*_*J*_, *k*_*d*_, and *difBetP* cannot be used as measurement of the dissimilarity between BBAs in this example.

Since *DismP* considers not only the distance but also the conflict between BBAs, the measurement based on the Hamacher T-conorm fusion rules provides a general method of the dissimilarity. However, it has dificiency as shown in *Example 3*.

*Example 3*. Let *m*_1_ and *m*_2_ be two BBAs on the same frame of discernment *Θ* = {*A*_1_,*A*_2_,…,*A*_20_}. For notation conciseness 1, 2, and so forth have been used to denote *A*_1_,*A*_2_, and so forth in the frame. The two pairs of BBAs are shown as

1st Pair:

$$m_{1}:\;m_{1}(2,3,4)={0.05,m}_{1}(7)=0.05,m_{1}(\Theta )=0.1,m_{1}(\Delta )=0.8$$

$$m_{2:}\;m_{2}(1,2,3,4,5)=1$$

**Fig 2:**
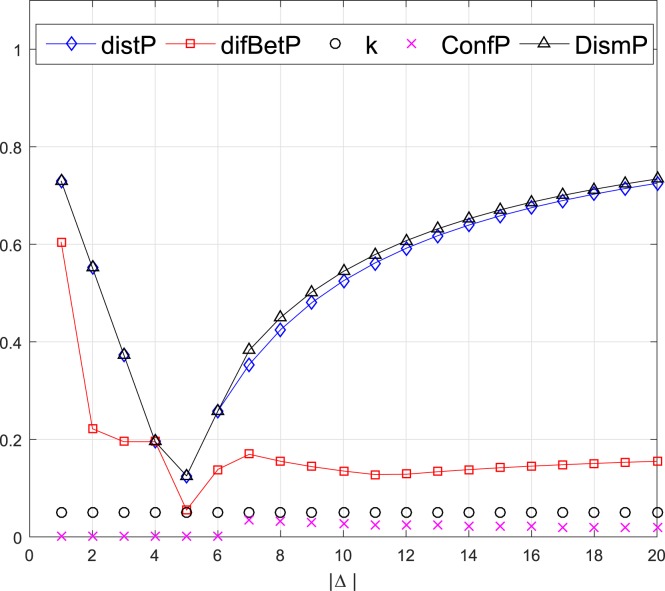
Comparison of dissimilarity measures of the 1st pair evidence.

**Fig 3:**
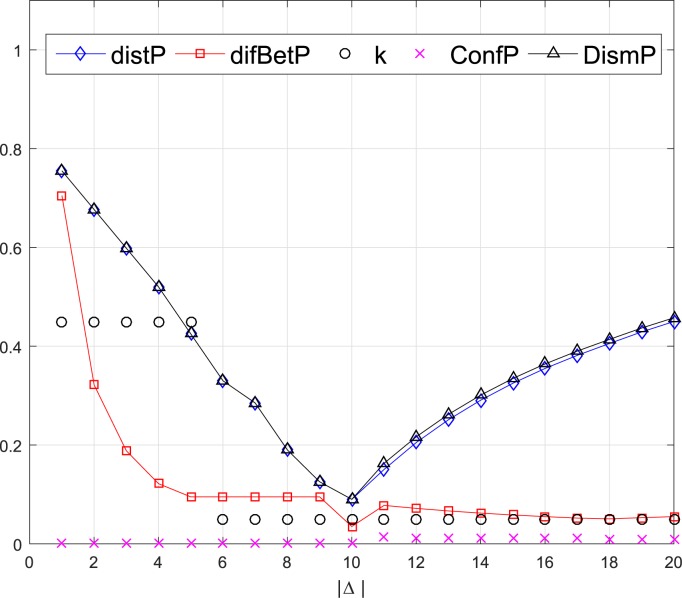
Comparison of dissimilarity measures of the 2nd pair evidence.

2nd Pair:

$$m_{1}:\;m_{1}(2,3,4)={0.05,m}_{1}(7)=0.05,m_{1}(\Theta )=0.1,m_{1}(\Delta )=0.8$$

$$m_{2}:\;m_{2}(1,2,3,4,5)=0.5,\;m_{2}(6,7,8,9,10)=0.5$$

where the Δ is a subset of *Θ*. This example considers 20 cases of the subset Δ, which increases by adding a new element at each case from Δ = {1} to Δ = {1,2,…,20}. The comparison of the dissimilarity measurements between *m*_1_ and *m*_2_ of the two pairs are shown in Figs 2 and 3 respectively.

From Figs 2 and 3, it can be seen that the *distP* and *DismP* are very close and follow the same trend, since the *DismP* are mainly decided by the *distP* and *ConfP*. As calculated in Eq (15), the maximal pignistic probabilities in both *m*_1_ and *m*_2_ always have intersection. So *ConfP* is small when the Δ increases from {1} to {1,2,…,20}. However, it is quite distinct situation of the two pairs of BBAs. In the 1st pair, both BBAs distribute their major belief to the same elements when the cases from 1 to 6, which cause that the *ConfP* keeps 0. This is reasonable as the *m*_2_ only has one focal element *m*_2_(1,2,3,4,5) = 1 which corresponds to classical conflict coefficient *k*. In the 2nd pair, *m*_2_ has two equal focal elements *m*_2_(1,2,3,4,5) = 0.5 and *m*_2_(6,7,8,9,10) = 0.5. As the case from 1 to 5, there should be a notable dissimilarity between *m*_1_ and *m*_2_, which is shown as *k* in Fig 3. Nevertheless, the pignistic probability transformation divides the belief equally to each single proposition as ${BetP}_{m_{2}}(1)={BetP}_{m_{2}}(2)=\cdots ={BetP}_{m_{2}}(10)=0.1$, indicating that the *ConfP* considers the dissimilarity as 0. Therefore, the dissimilarity measures of *ConfP* and *DismP* are illogical in this situation. Although the classical conflict coefficient *k* could depicts the dissimilarity from cases 1 to 5, it cannot reflect the variety of divergence degree as the case increases. Neither does the *difBetP*.

## Combining belief function with a new weighting factor

### 4.1 A modified dissimilarity measure

In this section, a modified dissimilarity measure is proposed which is based on the Hamacher T-conorm fusion rules to describe the dissimilarity between BBAs. The dissimilarity measurement based on Hamacher T-conorm rules satisfy two important properties of commutativity and monotonicity. The commutativity could ensure that the dissimilarity matrix is symmetrical and no matter the fusion order of two evidences is, their dissimilarity is coincident. The monotonicity provides that dissimilarity measurement has single variation trend in a specific interval, which is easy to compare the dissimilarity between evidences.

*Definition 11*. Let *m*_1_ and *m*_2_ be two BBAs on the same frame *Θ*. The modified dissimilarity measure is defined as

$${MDismP}_{m1}^{m2}≜T({distP}_{m_{1}}^{m_{2}},k_{m_{1}}^{m_{2}})=\frac{{distP}_{m_{1}}^{m_{2}}+k_{m_{1}}^{m_{2}}}{1+{distP}_{m_{1}}^{m_{2}}∙k_{m_{1}}^{m_{2}}}$$ (18)

where $k_{m_{1}}^{m_{2}}$ is the classical conflict coefficient.

$$k_{m_{1}}^{m_{2}}=\sum_{A_{i}\cap A_{j}=\emptyset }m_{1}(A_{i})m_{2}(A_{j})$$ (19)

The modified dissimilarity still satisfies the basic properties of commutativity and monotonicity:

(1) Commutativity:

MDismP(x,y)=MDismP(y,x) (20)

(2) Monotonicity:

$$0\leq MDismP(x,y)\leq MDismP(x^{'},y)\leq MDismP(x^{'},y^{'})\leq 1$$ (21)

where 0 ≤ *x* ≤ *x*′ ≤ 1 and 0 ≤ *y* ≤ *y*′ ≤ 1.

The modified dissimilarity measurement consists of a distance coefficient and a conflict coefficient between two evidences. As both the distance coefficient and the conflict coefficient lie in [0,1], the modified measurement is larger than its either components. The evidences have large distance and high conflict with the majority of other evidences would have a larger dissimilarity measurement and vice versa.

It is obvious that the modified dissimilarity measure replaces the conflict coefficient ${ConfP}_{m_{1}}^{m_{2}}$ with $k_{m_{1}}^{m_{2}}$. The ${ConfP}_{m_{1}}^{m_{2}}$ implies that the main conflict results from discordant propositions which are strongly supported by two BBAs respectively. However, the ${ConfP}_{m_{1}}^{m_{2}}$ cannot handle the situation that one BBA has several propositions with equal belief. As we see it, the conflict coefficient should involve all conflicts existed between BBAs no matter how small the extent of conflicts is. Furthermore, the *MDismP* not only maintains good features but also makes up for shortcomings of *DismP*.

**Fig 4:**
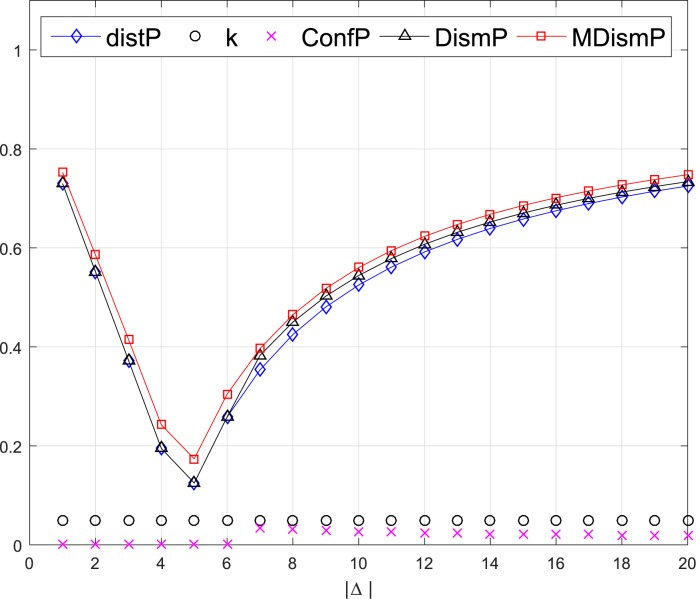
Comparison of dissimilarity of the 1st pair evidence.

**Fig 5:**
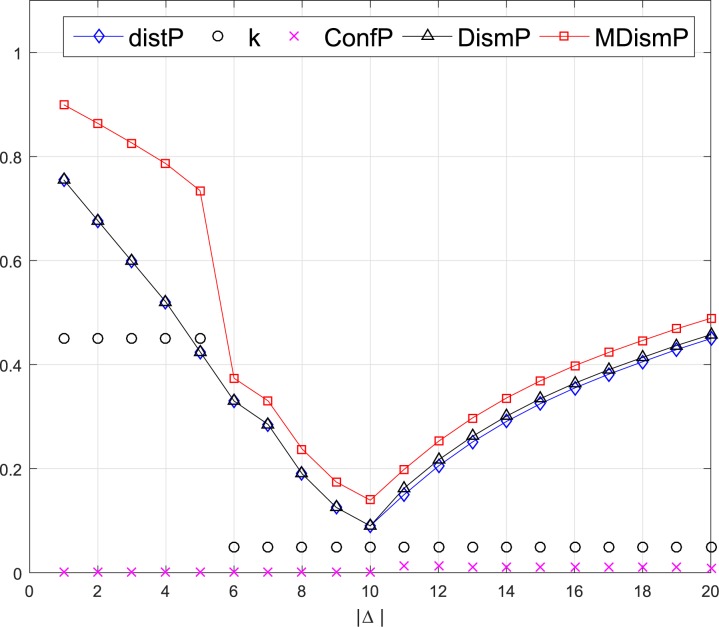
Comparison of dissimilarity of the 2nd pair evidence.

*Example 4*. Considering two pairs of BBAs from *Example 3* with the proposed dissimilarity measure of *MDismP*, the results are plotted in Figs 4 and 5.

For the results of the 1st pair illustrated in Fig 4, the *distP*, *DismP* and *MDismP* are identical. The lines show a variation tendency from a high dissimilarity when Δ = {1} to the minimum dissimilarity when Δ = {1,2,3,4,5} and increase again as Δ includes more elements. This is because the *m*_2_ only has one proposition and when Δ = {1,2,3,4,5}, the propositions with the maximum belief of two BBAs are accordant. In Fig 5, when *m*_2_ has two propositions with equal belief value, the results are different. The *MDismP* has a bigger value than the *distP* and *DismP* when Δ from {1} to {1,2,3,4,5}. The dissimilarity value of *MDismP* is near 0.8 before cases 6, which means the two BBAs are incompatible with each other. But the dissimilarity values of *distP* and *DismP* are less than 0.6, which seems unreasonable. Based on the analysis of the above examples, a conclusion can be drawn that the modified dissimilarity measure *MDismP* can efficiently reflects the degree of dissimilarity between BBAs.

### 4.2 Weighting factors

In this section, we propose a novel method to determine the weighting factors among BBAs based on the modified dissimilarity measure and Deng entropy.

The weight determinations are based on the principle that if an evidence is supported by greater number of evidences, this piece of evidence should be more important and have large effect on the final combination results. Moreover, if an evidence has considerable information, it also should be weighted more [37].

Suppose *N* evidences {*m*_1_,*m*_2_,…,*m*_*N*_} are in the same frame of discernment *Θ* and the weight of each evidence is made up of the degree of similarity and information volume. The similarity degree *Sim*_*i*,*j*_ of *m*_*i*_ and *m*_*j*_ is defined as

$${Sim}_{i,j}=1-MDismP(m_{i},m_{j})$$ (22)

the mutual similarity degree matrix S_*N*×*N*_ is then defined as

$$S_{N\times N}=\left[\begin{array}{cccc} 1 & {Sim}_{1,2} & \cdots & {Sim}_{1,N}\\ {Sim}_{2,1} & 1 & \cdots & {Sim}_{2,N}\\ \vdots & \vdots & \vdots & \vdots \\ {Sim}_{N,1} & {Sim}_{N,2} & \cdots & 1 \end{array}\right]$$ (23)

The S_*N*×*N*_ is symmetrical and *Sim*_*i*,*j*_ = *Sim*_*j*,*i*_ means that the similarity between two evidences fulfills the commutativity. The diagonal element is 1 means that an evidence is totally similar with itself. The similarity degree matrix helps give an insight into the agreement between evidences, and the weighting factor of dissimilarity could be obtained based on the similarity degree matrix.

The weighting vector *W*^*dis*^ of each evidence is associated with the eigen vector of the maximal positive eigen value λ_*max*_, that is λ_*max*_ ∙ *W*^*dis*^ = S_*N*×*N*_ ∙ *W*^*dis*^. The evidence with the largest weight is deemed to be the most important evidence and the weight of each evidence is revised as

$$\omega_{i}^{dis}=\frac{W_{i}^{dis}}{max(W_{i}^{dis})}\;i=(1,2,\ldots ,N)$$ (24)

the information volume of each evidence is measured by Deng entropy and can be calculated by Eq (16). After Deng entropy of each evidence is processed, the weights of information volume of evidence are obtained by

$$\omega_{i}^{Deng}=\frac{E_{d}(m_{i})}{max\;E_{d}(m_{i})}\;i=(1,2,\ldots ,N)$$ (25)

then the weight of each evidence based on the proposed method is defined as

$$\omega_{i}=\frac{\omega_{i}^{dis}+\omega_{i}^{Deng}}{\sum_{i=1}^{N}\left(\omega_{i}^{dis}+\omega_{i}^{Deng}\right)}\;i=(1,2,\ldots ,N)$$ (26)

The final weighting factor is measured by the weighting factor of dissimilarity measurement and the weighting factor of evidence information. The two weighting factors describe the final weight from two aspects: the factor of dissimilarity measurement depicts the mutual degree of deviation of an evidence with other evidences; the factor of evidence information illustrates the relative amount of information of an evidence compared to others. The final weighting factor is more thorough than the other weighting (discounting) factors which just depend on the dissimilarity measurement. With the weighting factor of information volume, the new weighting factor still hold the capability to avoid fusion error caused by the single information source failure.

### 4.3 Combination of evidences

The weighted average [29] and discounted [36] methods are two kinds of approaches that have been proposed for combining the evidences. The discounted method distributes the remaining mass value of the discounted mass to the universal set *Θ*, but this would result in more uncertainty. As the discounted methods need to combine the evidences one by one, the calculation will be time consuming and have a low convergence when a large amount of evidences need to be combined. The weighted average method would reinforce each other if the evidences are concordant and would weaken each other if the evidences are in conflict. The belief of propositions after combination will remain distinct from each other. In addition, the weighted average method is easily computational and more reliable and rational. Hence, the weighted average method is applied in this article.

The evidence generated by weighted average method is

$$EWA(m)=\sum_{i=1}^{N}\omega_{i}m_{i},\;i=(1,2,\ldots ,N)$$ (27)

For *N* original evidences, we should combine the new evidence *EWA*(*m*) for *N* − 1 times. In this section, an example of target recognition [36] is presented to show some behaviors of the existing method as well as the proposed method.

*Example 5*. Let *m*_1_, *m*_2_, *m*_3_, *m*_4_ *and m*_5_ be five BBAs on the same frame of discernment *Θ* = {*A*_1_,*A*_2_,*A*_3_} as shown in Table 1. The combination results obtained with the proposed methods are shown in Table 2. The convergence is shown in Table 3. $m_{1}^{i}$ means fusion of evidences *m*_1_,*m*_2_,…,*m*_*i*_, that is $m_{1}^{i}=m_{1}⨁m_{2}⨁\ldots ⨁m_{i}$.

**Table 1:** BBAs of five evidences.

	*m*_1_	*m*_2_	*m*_3_	*m*_4_	*m*_5_
***A***_**1**_	0.8	0.4	0	0.3	0.45
***A***_**2**_	0.1	0.2	0.95	0.2	0.1
***A***_**3**_	0	0.1	0.05	0.25	0
{***A***_**1**_,***A***_**2**_}	0	0.3	0	0.2	0
{***A***_**2**_,***A***_**3**_}	0	0	0	0	0.15
***Θ***	0.1	0	0	0.05	0.3

**Table 2:** Comparison results of different methods.

	$m_{1}^{2}$	$m_{1}^{3}$	$m_{1}^{4}$	$m_{1}^{5}$
***D* − *S* [36]**	**m(A**_**1**_**) = 0.8451** *m*(*A*_2_) = 0.0986 *m*(*A*_3_) = 0.0140 *m*(*A*_1,2_) = 0.0423	***m*(*A***_**2**_**) = 0.9948** *m*(*A*_3_) = 0.0052	***m*(*A***_**2**_**) = 0.9965** *m*(*A*_3_) = 0.0035	***m*(*A***_**2**_**) = 0.9971** *m*(*A*_3_) = 0.0029
**d_*J*_ [36]**	***m*(*A***_**1**_**) = 0.7659** *m*(*A*_2_) = 0.1166 *m*(*A*_3_) = 0.0294 *m*(*A*_1,2_) = 0.0881	***m*(*A***_**1**_**) = 0.6239** *m*(*A*_2_) = 0.2791 *m*(*A*_3_) = 0.0252 *m*(*A*_1,2_) = 0.0718	***m*(*A***_**1**_**) = 0.6858** *m*(*A*_2_) = 0.2645 *m*(*A*_3_) = 0.0146 *m*(*A*_1,2_) = 0.0315	***m*(*A***_**1**_**) = 0.7528** *m*(*A*_2_) = 0.2217 *m*(*A*_3_) = 0.0096 *m*(*A*_1,2_) = 0.0159
***DismP* [36]**	***m*(*A***_**1**_**) = 0.7503** *m*(*A*_2_) = 0.1196 *m*(*A*_3_) = 0.0319 *m*(*A*_1,2_) = 0.0957 *m*(*Θ*) = 0.0025	***m*(*A***_**1**_**) = 0.7157** *m*(*A*_2_) = 0.1598 *m*(*A*_3_) = 0.0308 *m*(*A*_1,2_) = 0.0913 *m*(*Θ*) = 0.0024	***m*(*A***_**1**_**) = 0.7670** *m*(*A*_2_) = 0.1655 *m*(*A*_3_) = 0.0194 *m*(*A*_1,2_) = 0.0477 *m*(*Θ*) = 0.0004	***m*(*A***_**1**_**) = 0.8254** *m*(*A*_2_) = 0.1424 *m*(*A*_3_) = 0.0120 *m*(*A*_1,2_) = 0.0198 *m*(*A*_2,3_) = *0*.*0002* *m*(*Θ*) = 0.0002
***Propsed*** ***Method***	***m*(*A***_**1**_**) = 0.7974** *m*(*A*_2_) = 0.1276 *m*(*A*_3_) = 0.0114 *m*(*A*_1,2_) = 0.0610 *m*(*Θ*) = 0.0026	***m*(*A***_**1**_**) = 0.8227** *m*(*A*_2_) = 0.1584 *m*(*A*_3_) = 0.0019 *m*(*A*_1,2_) = 0.0169 *m*(*Θ*) = 0.0001	***m*(*A***_**1**_**) = 0.8277** *m*(*A*_2_) = 0.1588 *m*(*A*_3_) = 0.0036 *m*(*A*_1,2_) = 0.0099	***m*(*A***_**1**_**) = 0.8651** *m*(*A*_2_) = 0.1249 *m*(*A*_3_) = 0.0048 *m*(*A*_1,2_) = 0.0046 *m*(*A*_2,3_) = 0.0004 *m*(*Θ*) = 0.0001

**Table 3:** The convergence of the proposed method.

***Fusion time n***	$m_{1}^{2}$	$m_{1}^{3}$	$m_{1}^{4}$	$m_{1}^{5}$
***n* = 1**	*m*(*A*_1_) = 0.7974 *m*(*A*_2_) = 0.1276 *m*(*A*_3_) = 0.0114 *m*(*A*_1,2_) = 0.0610 *m*(*Θ*) = 0.0026	*m*(*A*_1_) = 0.7160 *m*(*A*_2_) = 0.2149 *m*(*A*_3_) = 0.0115 *m*(*A*_1,2_) = 0.0553 *m*(*Θ*) = 0.0023	*m*(*A*_1_) = 0.6356 *m*(*A*_2_) = 0.2424 *m*(*A*_3_) = 0.0442 *m*(*A*_1,2_) = 0.0748 *m*(*Θ*) = 0.0029	*m*(*A*_1_) = 0.6158 *m*(*A*_2_) = 0.2247 *m*(*A*_3_) = 0.0555 *m*(*A*_1,2_) = 0.0676 *m*(*A*_2,3_) = 0.0169 *m*(*Θ*) = 0.0195
***n* = 2**		*m*(*A*_1_) = 0.8227 *m*(*A*_2_) = 0.1584 *m*(*A*_3_) = 0.0019 *m*(*A*_1,2_) = 0.0169 *m*(*Θ*) = 0.0001	***m*(*A***_**1**_**) = 0.7523** *m*(*A*_2_) = 0.2066 *m*(*A*_3_) = 0.0131 *m*(*A*_1,2_) = 0.0279 *m*(*Θ*) = 0.0002	*m*(*A*_1_) = 0.7292 *m*(*A*_2_) = 0.2051 *m*(*A*_3_) = 0.0270 *m*(*A*_1,2_) = 0.0297 *m*(*A*_2,3_) = 0.0055 *m*(*Θ*) = 0.0035
***n* = 3**			*m*(*A*_1_) = 0.8277 *m*(*A*_2_) = 0.1588 *m*(*A*_3_) = 0.0036 *m*(*A*_1,2_) = 0.0099	***m*(*A***_**1**_**) = 0.8086** *m*(*A*_2_) = 0.1654 *m*(*A*_3_) = 0.0118 *m*(*A*_1,2_) = 0.0120 *m*(*A*_2,3_) = 0.0016 *m*(*Θ*) = 0.0006
***n* = 4**				***m*(*A***_**1**_**) = 0.8651** *m*(*A*_2_) = 0.1249 *m*(*A*_3_) = 0.0048 *m*(*A*_1,2_) = 0.0046 *m*(*A*_2,3_) = *0*.*0004* *m*(*Θ*) = 0.0001

As can be seen from Table 2, the D-S combination rule (without the discounting or weighted average process) concludes that the proposition *A*_2_ is almost be regarded as the target. The result is unreasonable and counter-intuitive since majority of evidences distribute the major belief to proposition *A*_1_ and just one evidence *m*_3_ give its major belief to *A*_2_. Such unexpected behavior is solved by using the discounting and weighted average method to lessen the influence of the evidences which is dissimilar with the other evidences. The larger the dissimilarity of an evidence, the larger discount it will have. However, one also sees that the process of the proposed method as the dissimilarity measure to determine the weight of each evidence generates a more specific and reliable result than the process of discounting factors based on *d*_*J*_ and *DismP*. In addition, the $m_{1}^{3}$ is larger than the $m_{1}^{2}$ of *m*(*A*_1_) of the proposed method, which is completely opposite of *d*_*J*_ and *DismP* when the *m*_3_ assign its major belief to *A*_2_. What caused this is that the proposed new weighting factor weakens *m*_3_ twice by the weighting factor of dissimilarity and Deng entropy. For *d*_*J*_ and *DismP*, the discount factor weakens *m*_3_ once, which overcomes the defect raise by the classical combination rule. Besides, the evidence *m*_3_ has less information when compared to other evidences. So, weighting factor of Deng entropy further weaken the evidence *m*_3_ on the basis of the weighting factor of the dissimilarity factor. The larger $m_{1}^{3}$ of *m*(*A*_1_) of the proposed method reflects the proposed new weighting factor has a great capability of anti-interference. Furthermore, the proposed method has a better performance of convergence than the other methods. As can be seen from Table 3, the series results of proposed method achieve the belief level of other methods before the completion of the fusion process. For example, the three times fusion results of $m_{1}^{5}$ by the proposed method achieves 0.8 which achieves belief level to final result of *DismP* 0.8254, indicating that the proposed method provides faster convergence and is less computationally intensive. In the end, the great difference in *m*_3_, where almost all belief is given to *A*_2_, is caused by that evidence *m*_3_ may be especially sensitive to the typical characteristics of the kind of *A*_2_. The results in Table 2 show that the *A*_2_ with the second largest belief that is much larger than other propositions except *A*_1_. Therefore, *A*_2_ could be seen as a potential identification results as well.

This work provides a modified dissimilarity measure and evidence information volume measure to determine the weighting factors of evidences involved in the fusion process. The modified dissimilarity measure includes both the distances and all conflicts between evidences and the evidence information volume of each evidence is measured by Deng entropy. Compared with other methods, the proposed method gives a more specific and faster aggregated result. In addition, it is an efficient method in some decision-making applications.

## Identification of major land use activities contributing to river pollution: An application

In this section, the proposed method is applied to a case study in identifying the major land use activities contributing to river pollution and the required data are collected by water quality monitors.

The Manahara River lies in the Kathmandu Valley, Nepal, with a watershed of 256 km^2^. The river originates from Manichud Lekh (ridge) at an elevation of 2352 m and has a length of 30 km. The river originates from a pristine forested region and flows through forest, rural, semi-urban, and urban areas. The water quality was monitored monthly in seven different sites (Sites 1–7) of the river by sensors. The data of Sites 1 to 7, respectively Salinadi, Sankhu, Brahmakhel, Bode, Sinamangal, Imadol, and Chyasal are obtained from [44]. The land use and anthropogenic activities in transition of different sites are given in Table 4.

**Table 4:** Land use and river activities in site transition.

Site	Land use and river activities
**1 to 2**	*A1*^*a*^, *A2*
**2 to 3**	*A2*, *A3*
**3 to 4**	*A2*, *A3*, *A4*
**4 to 5**	*A2*, *A3*, *A6*
**5 to 6**	*A2*, *A3*, *A5*, *A6*
**6 to 7**	*A6*

^a^Note: *A1*: Forest, *A2*: Agriculture, *A3*: Bathing, washing & cleansing, *A4*: Rural settlement (sparse), *A5*: Industries, *A6*: Urban settlement (dense).

The water quality of the river during the low flow months in 2006 is given in Table 5. Water qualities are good in upstream and bad in downstream regions and varied gradually from Site 1 to 7. Each monitor records the amount of each chemical element in river when the water stream flow past each site. The actual variation amount of chemical element in each site should only consider the difference with the previous site. The chemical elements criteria in Table 5 could be divided into benefit criteria and cost criteria.

**Table 5:** Water quality of Manahara river in different sites.

Month	Site	DO^a^, mg/L	BOD mg/L	Free CO2, mg/L	TA mg/L	Cl, mg/L	NO3-N, mg/L	PO4 -P, mg/L	NH3 -N, mg/L	EC, uS/cm	TDS, mg/L
**February**	1	9.2	1.2	7.1	26	6.3	0.18	0.1	0.07	60.33	39.9
	2	9.2	1.3	9.2	39	6.7	0.32	0.2	0.2	71	47.1
	3	9.1	6.8	12.5	39	7.2	0.64	0.3	0.26	82	54.4
	4	8.5	14.9	17.8	67	9.0	0.72	0.4	0.61	94	62.4
	5	6.2	27.5	48.3	103	22.2	0.83	0.9	1.16	281.33	186.4
	6	2.2	154.9	106.8	352	57.6	2	4.2	4.42	925	613.0
	7	0	155.0	107.5	379	71.6	3.37	5.3	4.62	957.73	667.0
**March**	1	9.6	1.2	6.2	25	7.5	0.19	0.1	0.08	58.33	38.6
	2	8.9	1.8	6.9	31	8.4	0.35	0.2	0.22	70	46.4
	3	7.9	8.7	13.4	40	9.0	0.7	0.3	0.28	81.67	54.1
	4	7.3	16.7	14.1	73	13.2	0.75	0.4	0.66	116.33	76.7
	5	5.8	36.9	32.6	151	38.7	0.88	1.1	1.21	405.33	267.5
	6	5.7	83.8	55.9	276	68.7	1.82	3.3	3.25	719.33	474.7
	7	2.5	85.3	59.5	277	78.5	3.26	3.4	8.84	796	474.7
**April**	1	8.6	1.8	8.5	32	6.2	0.18	0.1	0.07	49.67	32.8
	2	7.2	2.8	8.7	33	7.4	0.25	0.2	0.22	75.33	49.9
	3	6.8	9.8	9.2	37	8.6	0.6	0.2	0.24	78	51.8
	4	6.5	17.8	9.3	41	11.8	0.63	0.4	0.39	91	60.3
	5	4.3	39.3	17.3	63	26.5	0.79	1.3	1.32	192.67	127.3
	6	3.3	113.6	37.1	167	43.2	1.79	2.9	2.86	473	278.3
	7	2.0	149.2	77.0	279	48.4	3.09	3.2	3.22	544.67	359.0
**May**	1	7.2	2.16	8.6	32	9.3	0.2	0.1	0.07	58.33	38.4
	2	6.7	3.8	13.4	33	9.7	0.26	0.1	0.22	97.33	64.1
	3	6.2	10.5	13.9	41	10.9	0.62	0.3	0.25	114.33	75.3
	4	6.0	18.4	13.9	41	13.3	0.67	0.4	0.55	122.33	80.6
	5	2.8	56.2	13.9	62	17.4	0.75	1.2	1.15	181.33	119.5
	6	2.3	119.0	24.9	130	25.0	2.24	1.8	3.04	296	195.0
	7	1.8	167.3	25.2	139	29.3	3.83	1.8	3.1	389.33	256.6

^a^Note: DO: Dissolved oxygen, BOD: Biochemical Oxygen Demand, TA: Total Alkalinity, Cl: Chloride, EC: Electrical conductivity, TDS: Total Dissolved Solids.

The change in water quality from one site to next site is normalized by Eqs (28) and (29). For a benefit criterion,

$$N_{i}=\frac{X_{i}-X_{i+1}}{X^{max}-X^{min}}$$ (28)

where $\sum_{i=1}^{n}N_{i}=1$ and *X*^*max*^ is the maximum value and *X*^*min*^ is the minimum value of the criterion. For a cost criterion,

$$N_{j}=\frac{X_{j+1}-X_{j}}{X^{max}-X^{min}}$$ (29)

where $\sum_{j=1}^{n}N_{j}=1$ and *X*^*max*^ is the maximum value and *X*^*min*^ is the minimum value of the criterion.

For example, the amount of DO (mg/L) in February is 9.2, 2.2 and 0 record in site 1, 6 and 7 respectively. DO (mg/L) is a benefit criterion and the normalization of site 6–7 is as Eq (30) shows.

$$N_{6}=\frac{X_{6}-X_{7}}{X_{1}-X_{7}}=\frac{2.2-0}{9.2-0}=0.239$$ (30)

The criteria, except the DO, are all cost criteria. The normalized chemical criteria of February are shown in Table 6.

**Table 6:** Normalized chemical criteria in different sites of February.

Month	Site	DO, mg/L	BOD mg/L	Free CO2, mg/L	TA mg/L	Cl, mg/L	NO3-N, mg/L	PO4 -P, mg/L	NH3 -N, mg/L	EC, uS/cm	TDS, mg/L
**February**	1–2	0.002	0.000	0.021	0.037	0.006	0.044	0.019	0.029	0.012	0.011
	2–3	0.009	0.036	0.033	0.000	0.008	0.100	0.019	0.013	0.012	0.012
	3–4	0.066	0.053	0.053	0.079	0.027	0.025	0.019	0.077	0.013	0.013
	4–5	0.248	0.082	0.303	0.102	0.201	0.034	0.096	0.121	0.209	0.198
	5–6	0.436	0.828	0.583	0.705	0.543	0.367	0.635	0.716	0.717	0.680
	6–7	0.239	0.000	0.007	0.076	0.215	0.429	0.212	0.044	0.036	0.086

In Table 6, each chemical criterion is seen as an evidence. Therefore, there are ten evidences need to be fused to identify which site causes the major pollution. According to the proposed method, the results of the river water quality monitoring and identification of February are given in Table 7.

**Table 7:** Fusion results of February by using the proposed method.

Land use and river activities	*A2*	*A6*	*A1 A2*	*A2 A3*	*A2*, *A3*, *A4*	*A2*, *A3*, *A6*	*A2*, *A3*, *A5*, *A6*
**Fusion Result** **of February**	0.057	**0.615**	0.000	0.151	0.000	0.134	0.041

From the Table 7, it shows that the ***A6*** urban settlement (dense) is the major cause for the river pollution. All the fusion results are listed in Table 8.

**Table 8:** Results of the application of the proposed method.

Land use and river activities	Fusion Result			
	Feb	Mar	Apr	May
***A2***	0.057	0.074	0.171	0.179
***A6***	**0.615**	**0.770**	**0.619**	**0.606**
***A1* & *A2***	0.000	0.000	0.000	0.000
***A2* & *A3***	0.151	0.114	0.112	0.142
***A2*, *A3*, & *A4***	0.000	0.000	0.000	0.000
***A2*, *A3*, & *A6***	0.134	0.038	0.087	0.069
***A2*, *A3*, *A5*, & *A6***	0.041	0.001	0.005	0.002

Table 8 shows that the major source of water pollution in all months are related to A6, i.e. urban settlement (dense). This result is true and reasonable as the adjacent area of Site 6 and 7 is A6 that discharged untreated sewage directly to those sites [45, 46], that have the worst water quality with very high BOD, nutrients (NO3-N and PO4-P), NH3-N, EC, and TDS with very low dissolved oxygen. Untreated sewage has high BOD, nutrients (NO3-N and PO4-P), NH3-N, EC, and TDS with very low or almost zero dissolved oxygen [47]. However, the combination (A2, A3, & A6) in Site 4 to 5 and the combination (A2, A3, A5, & A6) in Site 5 to 6 also have urban settlement (A6), which contributed insignificantly because urban settlement covered only a smaller land cover, is far from the river, and sewage outfall is absent in Site 4 and lower in Site 5.

The combination (A2, A3, A5, & A6) also has an industry which contributed insignificantly because there is only one beverage industry and its wastewater is diluted by the river flow and/or the sampling time would have been different than the discharge of concentrated industrial wastewater. In addition, the contribution of organic load by agriculture is much lower than that of sewage outfall [48]. Due to this, agriculture (A2) contribution has resulted in an insignificant pollution. Furthermore, the organic load contribution by forest (A1) is lower [49, 50], due to which the combination (A2 & A3) has insignificant contribution too.

In the four months from February to May, the results are similar. This is because the rivers have low flow in these months with February being winter and March to May being the pre-monsoon season [47]. The low flow in a river results in a high concentration of pollutants due to the lack of dilution. Therefore, the results of the application of the proposed method in identifying water polluting land use activities match the actual situation.

## Conclusion

Solving the problem of conflicting information fusion is still under continuous discussion. In this paper, a combination approach of evidences with a new weighting factor has been proposed based on a modified dissimilarity measure and Deng entropy between BBAs. The new weighting factor contains two part: weight of similarity and weight of entropy. The weight of similarity describes the degree of mutual support between BBAs. The larger the dissimilarity with others, the less the weight of similarity of a BBA. The weight of entropy describes the information volume of each BBA. If a BBA has a larger entropy value, meaning it contains more information, the weight of entropy of that BBA would be larger.

After analyzing the features and limitations of the existed dissimilarity measures, a modified dissimilarity measure based on the Hamacher T-conorm fusion rules mixing the probabilistic-based distances with the all conflict between BBAs has been developed. Also, the weight of similarity is determined by dissimilarity measures. The information volume of a BBA is obtained by Deng entropy and is used in the determination of the weight of entropy.

The new weighting factor based on the modified dissimilarity and Deng entropy is presented and applied in the weighted average combining belief function. Several numerical examples analysis show that the proposed method not only obtains a more reasonable and specific result but also has a faster convergence rate. A real application of determining the major land use activities contributing to river pollution is implemented by the proposed method. Result shows that the urban settlement (dense) is the major source of water pollution. This new approach can be of great interest for decision makers in devising strategies to control water pollution and environmental management.
